# Genomic Analysis of Puerto Rican Hispanic/Latino Men with Prostate Cancer

**DOI:** 10.3390/cancers18071091

**Published:** 2026-03-27

**Authors:** Jamie K. Teer, Gilberto Ruiz Deya, Sol V. Pérez-Mártir, Jong Y. Park, Jose Oliveras, Julie Dutil, Jaime Matta

**Affiliations:** 1 Department of Biostatistics and Bioinformatics, H. Lee Moffitt Cancer Center and Research Institute, Tampa, FL 33612, USA; 2 Urology Residency Program, Department of Surgery, Ponce Health Sciences University, St. Luke’s Hospital, Ponce, PR 00717, USA; 3 Division of Clinical and Translational Cancer Research, Comprehensive Cancer Center of the University of Puerto Rico, San Juan, PR 00921, USA; 4 Department of Cancer Epidemiology, H. Lee Moffitt Cancer Center and Research Institute, Tampa, FL 33612, USA; 5 Public Health Program, Ponce Research Institute, Ponce Health Sciences University, Ponce, PR 00716, USA; 6 Department of Basic Sciences, Ponce Research Institute, Ponce Health Sciences University, Ponce, PR 00716, USA

**Keywords:** prostate cancer, genomics, Hispanic/Latino, somatic mutations, germline variants, gene fusions, Puerto Rican

## Abstract

**Background/Objectives:** Puerto Rican Hispanic/Latino (PR H/L) men experience a heightened incidence and mortality rate of aggressive forms of prostate cancer. The underlying causes of this increased disease burden likely include a complex interplay of socio-economic and biological factors. This pilot study leveraged the first cancer tissue biobank at a Hispanic-Serving Institution (Puerto Rico BioBank) and aimed to provide an initial description of the genomic features of prostate cancer in 35 PR H/L men. **Methods:** Whole-exome and RNA sequencing were performed on prostate adenocarcinoma tumor samples to investigate the genomic features associated with prostate cancer. **Results:** Our analysis suggests that mutation profiles and gene expression pattern differences are observed in this population and may be associated with disease aggressiveness and progression. Notably, mutations in *TP53* and *TMPRSS2-ERG* gene fusions, which are common in broader populations, were less prevalent in the PR H/L cohort. **Conclusions:** While this study contributes to the understanding of ethnicity-specific genetic factors in prostate cancer, underscoring the need for inclusive genomic studies, continued expansion to larger cohorts of patients under-represented in large genomic studies will be needed to more robustly characterize the full range of genomic features of prostate cancer. A broader understanding of the genomic features of prostate cancer in PR H/L men may lead to future opportunities for delivering more personalized prognoses and treatment options, helping to ensure that treatment advances and better outcomes are available to all patients.

## Simple Summary

This pilot study explored the genomic features of prostate cancer in Puerto Rican Hispanic/Latino men, a group disproportionately affected by this disease, in terms of incidence, mortality and aggressiveness. By examining tumors from 35 patients for specific genetic mutations and gene expression patterns using genomic sequencing, we aimed to investigate distinct genetic profiles that could influence the course of the disease and its treatment. Our initial findings suggest potential differences in genomic characteristics in this population, offering a future avenue for developing personalized treatments that could reduce the health disparities observed in these men. This study underscores the importance of including diverse ethnic groups in genomic studies to ensure a broad biological assessment of prostate cancer features informs future personalized cancer care for all patients.

## 1. Introduction

Prostate cancer (PCa) is a major health concern, particularly in elderly men. It is the second most frequent malignancy in men worldwide and the fourth most common globally [1,2,3,4]. In 2025, 313,780 new PCa cases will be diagnosed, and 35,770 PCa-related deaths will occur in the United States [5]. This makes PCa the second leading cause of cancer-related mortality among men in the US [6]. PCa is the most commonly diagnosed cancer in Hispanic/Latino (H/L) men in the US [7], and research indicates that they are more likely to present with higher-risk forms of the disease than other ethnic groups [8]. In Puerto Rican men, a subgroup of the H/L population, PCa is the leading cause of cancer-related mortality (19%) and incidence (38%) [9]. PCa presents significant health disparities among Puerto Rican and other Hispanic/Latino (H/L) populations, particularly in terms of mortality and access to care. Puerto Rican H/L men experience worse outcomes and higher prostate cancer-specific mortality rates than other Hispanic groups, highlighting the urgent need for more studies on the biology of PCa and targeted health interventions in this demographic group [10,11,12]. Non-genetic factors such as access to care [13], socioeconomic status [14], treatment delays [15], healthcare system and insurance coverage [13,16], lifestyle and dietary factors [14], comorbidities and metabolic disorders [13] and environmental exposures [17,18] all contribute significantly to these prostate cancer disparities.

Molecular and biological differences in prostate tumors that may contribute to health disparities in H/L patients are not well understood. Most genetic studies have predominantly focused on individuals of European ancestry, with H/L populations representing approximately 1% of the samples in cancer genome-wide association studies [19]. Historically, H/L populations have been underrepresented in cancer research, creating challenges in understanding the full scope of the genetic and other factors that influence PCa risk [20]. This lack of representation poses challenges in understanding the genetic factors influencing PCa risk among H/L men. The mutational profiles of Africans and African Americans, with whom Puerto Rican H/L share an ancestral heritage, provide early indications of the benefits of conducting PCa genomic studies in diverse populations.

Germline DNA sequencing of PCa patients is an emerging area of research that aims to unravel the genetic complexities of this prevalent malignancy. This innovative technique leverages high-throughput sequencing technologies to identify germline genetic susceptibility variants that significantly influence the risk and progression of PCa [21]. Notably, germline mutations in genes such as *BRCA1*, *BRCA2*, and *ATM* are linked to more aggressive forms of PCa, highlighting the importance of genetic testing for targeted therapeutic strategies [22,23]. Studies have shown that more than one-third of PCa risk can be attributed to germline factors, with pathogenic variants found in a significant proportion of patients, particularly those with advanced disease [24,25,26,27,28]. Moreover, research has shown that approximately 10% of men with metastatic PCa harbor germline mutations in DNA repair genes, emphasizing the need for genetic testing and personalized treatment approaches tailored to unique genetic profiles [26,29,30].

Tumor somatic mutations play a critical role in the pathogenesis, progression (aggressiveness), and treatment response of cancers. Compared to other tumors such as melanoma, PCa generally has a low tumor mutational burden [31,32]. The specific driver mutations found differ for localized primary PCa and metastatic castration-resistant PCa (mCRPCa) [33,34]. Somatic mutations in *BRCA1* and *BRCA2* and DNA damage response (DDR) pathways have been identified in PCa [35,36], and patient tumors with DDR mutations respond to PARP inhibitors [29]. Certain somatic mutations, such as those in the *MYC* oncogene, manifest early in PCa development and are linked to more aggressive forms of the disease [34,37]. Additionally, specific somatic mutations such as those in the androgen receptor gene (*AR*) have been linked to PCa aggressiveness and DNA repair dysregulation [38]. Hispanic/Latino men also have a greater chance of presenting with higher-risk localized PCa than non-Hispanic white men [8]. Tumors from H/L men may exhibit distinct mutation profiles compared to those from other ethnicities, as evidenced by a recent study showing different mutation rates and types among various racial groups. In a study of 1412 primary PCa tumors including an analysis of 465 genes, H/L men had higher alternation frequencies than non-H/L men in *TMPRSS2* and *CCNE1* [39].

Despite the high PCa mortality rate in Puerto Rican H/L men, no published genomic data are available for PCa tumors in this population. Consequently, there is an urgent need for more inclusive research that explores the genomic landscape and treatment responses in PR H/L men, which could ultimately lead to improved screening and PCa therapeutic strategies tailored to their unique needs [12]. Understanding the genomics of PCa tumors in PR H/L men will enable enhanced diagnosis, elucidate specific pathways to target for treatment, and identify clinically actionable mutations. This pilot study is the first to report the molecular characteristics of prostate adenocarcinomas in 35 H/L Puerto Rican men and an initial exploration of their association with selected clinicopathological variables. Given the known health disparities, our study aimed to elucidate the genomic factors contributing to PCa in Puerto Rican Hispanic/Latino men.

## 2. Materials and Methods

### 2.1. Recruitment and Clinicopathological Variables

PCa cases for this study were recruited through the standard protocols of the Puerto Rico Biobank of the Ponce Research Institute, which participates in the Oncology Research Information Exchange Network (ORIEN) through a collaboration with the H. Lee Moffitt Cancer Center, 1 of 17 contributing centers nationwide. Briefly, the study nurse or physicians obtained written informed consent from all study participants preoperatively. In addition to donating a tumor sample (formalin-fixed embedded paraffin (FFPE) blocks), participants completed a survey covering demographic data, history of diseases, and risk factors. All PCa cases were pathologically confirmed to be primary PCa. A trained data abstractor extracted clinicopathological variables from the patients’ records, including PSA, Gleason Score, and pathology grade (histologic assessment of tumor differentiation and cellular atypia as determined by a pathologist using tissue specimens), in accordance with the ORIEN data dictionary, consisting of over 250 data points, with follow-up every six months. For downstream analysis, patients were stratified into indolent or aggressive categories based on the Gleason score (GS): indolent (GS 6 and 7 (3 + 4)) and aggressive (GS 7 (4 + 3) and ≥8). Patients were also classified into five grade groups using their GS according to the NCCN Guidelines for Prostate Cancer (2025) (NCCN.org/guidelines). All participants were recruited by nurses or physicians in the clinical practice of Gilberto Ruiz-Deya, MD, at San Lucas Hospital in Ponce, Puerto Rico. Molecular profiling (whole exome sequencing and bulk RNA-sequencing) was performed using the ORIEN Avatar Program, as described below in the text.

### 2.2. Institutional Review Board

The study was conducted in accordance with the Declaration of Helsinki and was approved before the initiation of the study by the Institutional Review Board (or Ethics Committee) of the Ponce Research Institute (protocol 2101051235R001) and Moffitt Cancer Center (MCC protocol MCC21345).

### 2.3. Whole Exome Sequencing

Tumor samples were required to contain >30% tumor and were macrodissected to enrich the tumor tissue. DNA samples from tumors and matched normal tissues were subjected to Whole Exome capture using either the IDT xGen probe set supplemented with additional probes to provide double coverage of 440 actionable genes or the Agilent SureSelect XT HS2 Clinical Research Exome Kit. The samples were sequenced in a paired-end configuration using Illumina sequencers (Illumina, San Diego, CA, USA).

Sequence reads were trimmed using Cutadapt 1.8.1 [40] or AGeNT Trimmer 3.1.2 (Agilent, Santa Clara, CA, USA) to remove adapters and low-quality bases. Trimmed reads were aligned to the GRCh38 human reference genome using bwa 0.7.17-r1188 [41] and converted to BAM files using samtools 1.9 [42]. Duplicate read identification was performed using GATK 4.1.2 [43] or AGeNT CReaK 1.1.1 (Agilent, Santa Clara, CA, USA) and base quality recalibration was performed using GATK 4.1.2. Indel realignment was performed using GATK 3.8.1.

Somatic mutations were detected by comparing DNA sequence from the tumor and non-tumor germline samples from the same patient. Genetic variants unique to the tumor are determined to be somatic mutations. Multiple somatic mutation identification tools were used, and mutations identified by at least 2 methods were included in downstream analyses. Single nucleotide variant callers included MuTect 1.1.7 [44], Strelka 1.0.15 [45], Muse 1.0rc [46], SomaticSniper 1.0.5 [47], and Freebayes 1.3.1 [48]. The deletion and insertion mutation callers used were Mutect2 [49] (included in GATK-4.1.2), Strelka 1.0.15 [45], Strelka2 2.9.10 [50], Freebayes 1.3.1 [48], and Lancet 1.1.0 [51]. COSMIC v3 somatic mutation signatures were calculated using DeconstructSigs 1.9.0 [52].

Somatic copy number variants (CNVs) were detected by comparing depth of coverage and variant allele frequency in a tumor and non-tumor germline sample from the same patient; relative changes in these metrics are used to mathematically infer loss or gain of sequences across the genome. Sequenza 3.0.0 [53], ASCAT 3.1.2 [54] and CNVkit 0.9.10 [55] were used to identify candidate CNVs and each gene was assigned a CNV state (amplification, deletion, or wild type). Gene-level CNVs were included in downstream analysis if identified by ≥2 methods, excluding variants where the methods disagreed on direction (i.e., CNVs where one method identified an amplification and another identified a deletion in the same sample were excluded). Recurrent CNVs were observed in more than five samples in the same direction. The *AR* gene, located on chrX, was manually reviewed for copy-number changes given the variability in CNV representation on the sex chromosomes; no CNVs met the above criteria.

Germline variants were identified using GATK 4.1.2 HaplotypeCaller, GenotypeGVCFs, and VQSR. Variants with a genotype quality of at least 10, VQSR Tranche of PASS, and a variant allele frequency of at least 30% were retained for the analysis. Variants and mutations were annotated using ANNOVAR 06212012 [56].

### 2.4. RNA Sequencing

RNA libraries were prepared using the Illumina TruSeq RNA Exome kit with a strand-specific approach focused on transcriptome coding regions. The RNA samples were sequenced using Illumina sequencers. The sequences were aligned to the human reference genome GRCh38 using STAR 2.7.7a [57]. Gene expression was quantified using RSEM 1.3.0 [58]. Differential expression was determined using DESeq2 1.48.1 [59], accounting for the batch and removing genes with <10 reads in >10 samples. Genes with an absolute value of log2 fold change > 2 and Benjamini–Hochberg adjusted *p*-value < 0.05 were considered significantly differentially expressed. For plotting purposes, batches were corrected using Combat-seq (sva 3.38.0) [60] and the counts were normalized and log2 transformed. Gene fusions were identified using FusionCatcher 1.30 [61] or Arriba 2.5.1 [62] software. Candidate genes annotated using banned terms were excluded from the analysis.

### 2.5. Large External Datasets

Mutation data from large external datasets were extracted from cBioPortal: TCGA (Prostate Adenocarcinoma (TCGA, Cell 2015)) and MSK-IMPACT (Race Differences in Prostate Cancer (MSK, 2021)). To better match the PR H/L cohort, external cohorts were filtered to only include primary adenocarcinoma tumors with lower-grade Gleason Scores (<7, 3 + 4). When results were examined by race, self-reported race was used. Ethnicity was not available in the external datasets examined.

## 3. Results

### 3.1. Clinicopathological Characteristics of the Study Cohort

Table 1 provides an overview of the patient and tumor characteristics for the study cohort, along with breakdowns for the Whole Exome Sequencing (WES) subset and RNA-sequencing (RNA-seq) subset. 35 patients were included in the study and molecular profiling was attempted for all samples; Whole Exome Sequencing (WES) was successfully completed on 20 patients’ tumor/normal sample pairs and RNA sequencing (RNAseq) was performed on 34 patients’ tumor samples. All patients were Hispanic/Latino men who lived in Puerto Rico and were diagnosed with primary prostate adenocarcinoma. For the majority of patients (76.9%), tumors were treatment-naïve at the time of surgery. The age of the patients ranged from 53 to 73 years, with a mean age of 64 years in all groups.

The WES and RNA-seq subsets largely reflected the characteristics of the full cohort, suggesting that they are representative samples for further genomic and transcriptomic analyses. Overall, the majority of patients had T2 stage tumors (68.57%) and showed no lymph node involvement (N0) (91.43%). The Gleason grades (GG) were weighted towards indolent diseases, with 71.43% of the tumors having GG 1 or 2. The PSA level at diagnosis ranged from 0.0 to 26.0 nanograms per milliliter (ng/mL), with a mean of 6.60 ng/mL. Information on distant metastasis was missing for a significant portion of the cohort (65.71%).

### 3.2. Tumor Mutation Characteristics

**Figure 1:**
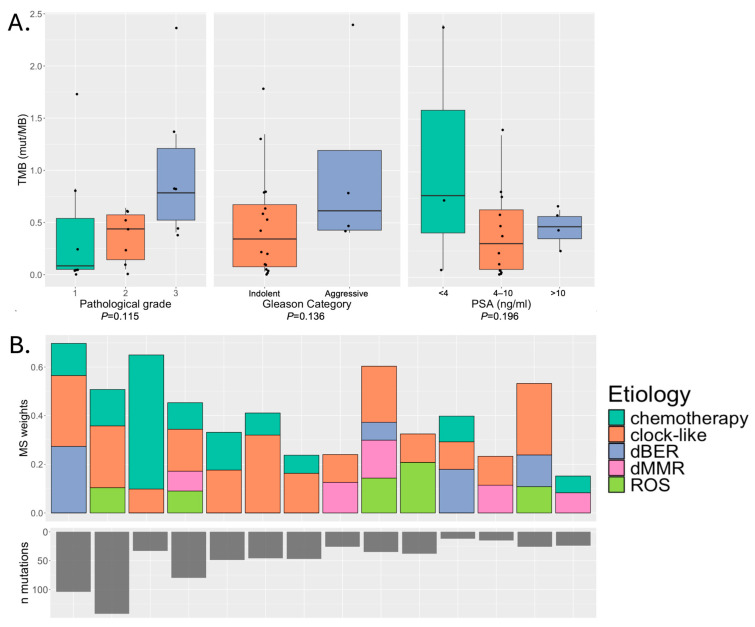
(**A**) Associations between tumor mutational burden (TMB) and pathological characteristics of the tumor (individual patient data displayed as points) and (**B**) commonly observed mutational signatures in patients with ten or more protein-altering mutations.

Tumor somatic mutation characteristics were examined using WES on tumor and matched normal samples from H/L patients with PCa. We observed a median of 77.5 total mutations per sample (range: 8–509) and a median of 26 protein-altering mutations per sample (range: 2–142). The median average depth of coverage in the tumor samples was 176 (range 78–552) and in the germline samples was 139 (range 72–316) (Supplemental Tables S1–S3). Tumor mutational burden (TMB), defined as the number of protein-altering somatic mutations per million bases sequenced (Mb), was calculated and compared after grouping the tumors according to their clinicopathological characteristics (Figure 1A). The median TMB was 0.44 (range 0.03–2.39 mutations per Mb). TMB was not significantly different across tumors based on indicators of aggressiveness, such as higher pathologist assigned grade and higher Gleason category (pathological grade *p* = 0.115, Gleason category *p* = 0.136; PSA *p* = 0.196).

Next, we explored the patterns of mutational signatures grouped by associated etiology. The upper panel of Figure 1B displays the normalized signature weights (NS weights), which indicate the relative contribution of different mutational processes to the overall mutational landscape of tumors. The etiologies include chemotherapy; clock-like signatures reflecting endogenous mutational processes that accumulate over time, often related to cell division; dBER (defective Base Excision Repair), indicative of deficiencies in the BER pathway; defective Mismatch Repair (dMMR), characteristic of defective DNA MMR, which leads to microsatellite instability; and Reactive Oxygen Species (ROS), linked to DNA damage caused by reactive oxygen species. The most prevalent signatures were those associated with aging (“clock-like” signatures), observed in 75% of the tumors and ranged from 0% to 31% NS weights (median = 11.8%). Some patients also showed evidence of chemotherapy-associated mutational signatures. Understanding TMB alongside mutational signatures is crucial, as it provides insights into the underlying mutational processes and potential therapeutic targets in these tumors.

### 3.3. Tumor Alteration Profiles

**Figure 2:**
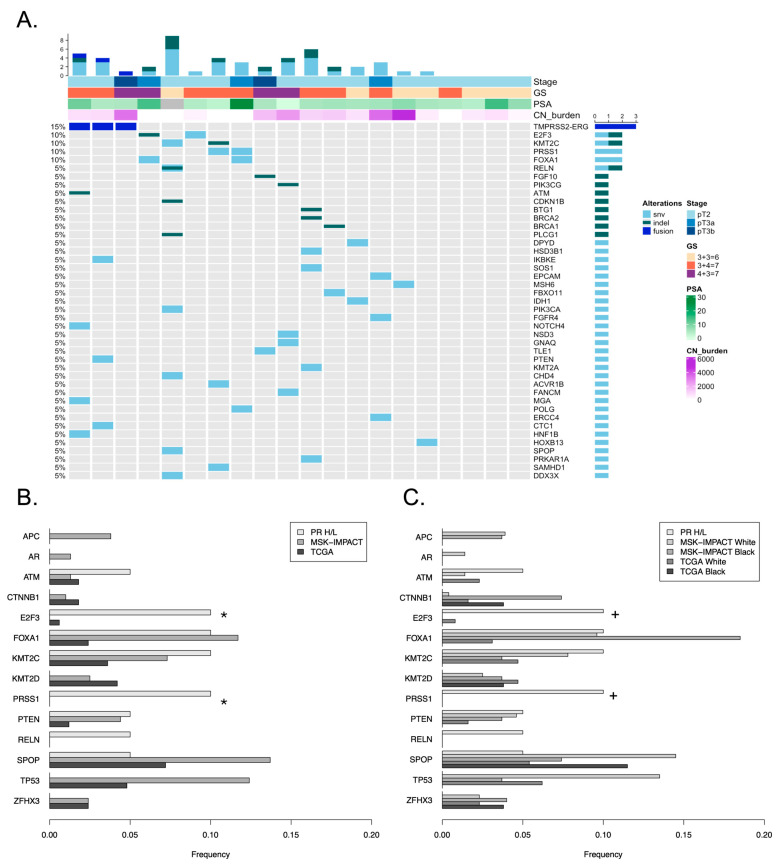
Somatic mutational landscape of tumors profiled using WES (n = 20). (**A**) Oncoprint representing the most frequently mutated OncoKB-annotated genes. (**B**) Comparison of the mutational frequencies in the Puerto Rico cohort with the Memorial Sloan Kettering (MSK) and TCGA Pan Cancer Atlas cohorts (primary tumors with Gleason Score < 7, 3 + 4). (**C**) Comparison as in b., showing frequency in MSK and TCGA by self-reported race. Fisher’s exact test *p*-value < 0.05 for PR/H/L vs. all groups noted with *; vs. White noted with +.

Next, we explored the mutation profiles of the most frequently mutated genes in OncoKB, a Memorial Sloan Kettering precision oncology knowledge base [63,64]. Few recurrent mutations were observed in our cohort; only *E2F3*, *KMT2C*, *PRSS1*, and *FOXA1* were mutated in more than one patient (Figure 2A). As 80% of the PR H/L cohort tumors had lower Gleason grades 1–2, we compared mutation frequencies with those observed in lower Gleason grade primary adenocarcinoma tumors (Gleason < 7, 3 + 4) from two large studies: MSK-IMPACT prostate adenocarcinoma (n = 315 primary low-grade prostate tumor; targeted sequencing) [65] and The Cancer Genome Atlas (n = 167 normal/primary prostate tumor; whole exome sequencing) [35]. The mutation frequencies of several commonly mutated PCa genes were lower in the PR H/L cohort than in MSK-IMPACT and TCGA, including *SPOP* (5% vs. 13.7% and 7.2%) and *TP53* (0% vs. 12.4% and 4.8%) (Figure 2B) although these differences were not significant (Fisher’s exact test *p* ≥ 0.05). No mutations were observed in the *AR* gene. Mutations in other genes observed in H/L PCa (*TMPRSS2*, *CCNE1*, *KRAS*) were not observed in this PR H/L cohort. Mutation frequencies of *E2F3* and *PRSS1* were significantly higher (Fisher’s exact test *p* < 0.05) in the PR H/L compared to both MSK-IMPACT and TCGA, although the number of samples with the mutation was low. Mutation frequencies compared to the full primary tumor external cohorts including Gleason-high tumors are shown in Supplemental Figure S1. The sequence depth of coverage for these genes in our study was high (100% of coding bases were covered by at least ten reads for all genes except *PRSS1*, which was covered 95% due to lack of target coverage of an alternate exon unique to one transcript) (Supplemental Figure S2).

Comparisons with external datasets are often complicated by a higher propotion of White patients relative to the general population. We examined the mutation frequency in external populations by self-reported race to explore possible differences in mutation frequencies (Figure 2C). The mutation frequency of *TP53* is lower in tumors from Black patients than in those from White patients in TCGA and MSK-IMPACT, although this was not significant (Fisher’s exact test *p* ≥ 0.05). This is potentially interesting given the higher proportion of African genetic similarity in Puerto Rican individuals when compared to Central American Hispanic/Latino individuals. Conversely, mutations in *CTNNB1* and *ZFHX3* were not observed in PR H/L patient tumors, but were observed more frequently in tumors from Black patients than in those from White patients in TCGA and MSK-IMPACT. The other genes did not exhibit consistent patterns in external cohorts. We note that the number of non-White patients in all cohorts was small, and greater numbers of samples are needed to more robustly evaluate population-level mutation differences.

We further examined somatic copy number alterations in the commonly mutated PCa genes. Only one PR H/L tumor sample (Gleason aggressive) exhibited a heterozygous *TP53* deletion. In contrast to the MSK-IMPACT and TCGA studies, which reported recurrent *PTEN* homozygous deletions (4.4% and 10.2%, respectively) and truncating mutations (3.5% and 0.6%, respectively), we did not observe any deletions, although we did observe three samples with amplification. We observed only one tumor (Gleason indolent) with a *PTEN* mutation, which was predicted to be in a splice site. Somatic alterations observed in *SPOP* in the TCGA study were primarily mutations in several recurrent positions; however, we observed an amplification in two samples in addition to a single point mutation (p.F102S, which was a recurrent position in the TCGA study). Several mutations commonly observed in other tumor types were observed in the PR H/L cohort: an *IDH1* R132H mutation in one patient’s indolent tumor (compared to 0.3% in MSK-IMPACT and 1.2% TCGA) and *PIK3CA* C420R observed in another patient’s indolent tumor (compared to 0% in MSK-IMPACT and 0% in TCGA). We observed other known driver mutations in *IDH1* and *PIK3CA* in MSK-IMPACT and TCGA, suggesting that alterations in these genes may contribute to PCa.

Recurrent CNVs (observed in more than five samples in the same direction) were identified in 163 genes, with alterations observed in 6–8 samples each (Supplemental Figure S3, Supplemental Table S4). These genes clustered together (mostly <10 Mb) in a consistent direction, suggesting larger events. Deleted gene clusters were grouped at the q end of chromosome 20 (20q13.33, 61 genes), p end of chromosome 11 (11p15.5, 23 genes), q end of chromosome 14 (14q32.33, 14 genes) and towards the q end of chromosome 19 (q13.42, 12 genes including *LILR* members). Amplified gene clusters were grouped at chr6p22.1 (16 genes in a histone cluster region), chr6p21 (24 genes in the MHC region), towards the q end of chromosome 8 (chr8q24.22–24.3, 7 genes) and chr12q13.13 (4 genes). The concentration of deletions towards the chromosomal ends suggests that telomere shortening may occur in some PR H/L tumors. To determine the potential biological relevance, we examined the correlation between the CNV state (DEL = 1, WT = 2, AMP = 3) and gene expression levels. Only 2 genes showed a positive significant correlation (Kendall *p* < 0.05, uncorrected): *AL662884.4* and *HIST1H2AJ* (Supplemental Figure S4).

Somatic mutations in DNA damage repair genes are of increasing interest because of newer clinical interventions targeting deficiencies in DNA damage repair such as PARP inhibitors. We examined genes associated with Homologous Recombination Repair (HRR) and Nucleotide Excision Repair (NER) pathways (Supplemental Table S5). HRR gene analysis identified somatic truncating mutations in *ATM* (a frameshift insertion at p.I1249) in one patient’s tumor and *BRCA2* (a frameshift insertion at p.Q1782) in another patient’s tumor, both of which were indolent. Truncating mutations were observed in these genes in both MSK-IMPACT and TCGA at a frequency of <10%. We observed recurrent CNV deletions in *XRCC3* (two indolent tumors) and recurrent amplifications in *RPA1* (two aggressive, one indolent tumors), *RAD54B*, *RAD51B*, *NBN*, *BRCA1*, and *ATR* (each observed in one aggressive and one indolent tumor) (Supplemental Figure S5A). The tumor sample with a *BRCA2* frameshift insertion also contained a *BRCA2* copy number deletion. Overall, 10/20 (3/4 Gleason Aggressive) tumors had alterations in HRR genes. No truncating mutations were observed in the NER genes. Three tumors had deletions in *POLE*, four tumors had amplifications in *GTF2H4*, and three tumors had amplifications in *RPA1* and *LIG3* (Supplemental Figure S5B).

### 3.4. Known Fusions in Prostate Cancer

Gene fusions involving E26 transformation-specific (ETS) family transcription factors are common drivers of PCa. Gene fusion detection was performed using RNAseq data available from 34 PR H/L tumors. We observed four tumors with *TMPRSS2-ERG* fusions (12%), compared to 34% (107/315) of tumors with *TMPRRS2* or *ERG* structure variants in the MSK-IMPACT cohort (*p* = 0.007, Fisher’s exact test) and 57% (95/167) of tumors with ETS family gene fusions in the TCGA study (*p* < 0.001, Fisher’s exact test) (Figure 2A, one patient with a fusion is not shown due to lack of WES data). We had observed a *TMPRSS2-ETV1* fusion in a single sample; however, following a manual review, we determined that this fusion resulted in a frameshift in ETV1, likely rendering it non-functional. We did not include this frameshifting *ETV1* fusion in downstream analyses. No other fusions of ETS genes were observed. We also observed amplification of ETS genes: *ERG* (1 tumor without ETS fusions) and *ETV4* (2 tumors, 1 without ETS fusions). In total, 5/20 (25%) patients had amplifications or fusions involving ETS genes, which is lower than that observed in other large studies. Similar to TCGA, mutations in *SPOP* were mutually exclusive with alterations in the ETS genes in this cohort. Fusions and amplifications involving ETS genes were not significantly different between the Gleason indolent and aggressive categories. Interestingly, although *ERG* fusions had higher *ERG* gene expression, the sample with *ERG* amplification did not (Supplemental Figure S6). Two additional tumors without detected fusions had higher *ERG* expression levels similar to those of tumors with fusions, suggesting potential *ERG* activation in two additional PR H/L tumors.

### 3.5. Germline Variants in DNA-Damage Repair Genes

Loss-of-function germline variants in DNA damage repair genes are known to increase cancer susceptibility and have been increasingly observed in PCa patients. We examined germline variants in 26 homologous recombination repair (HRR) and 30 nucleotide excision repair (NER) genes (Supplemental Tables S5 and S6) that were predicted to result in protein truncation in PR H/L patient tumors. We focused on variants that were not commonly observed (<1%) in the Exome Aggregation Consortium, NHLBI Exome Sequencing Project, or 1000 Genomes Project. Only one patient had a heterozygous truncating mutation in *BRCA2*: p.K3326* (rs11571833). Truncating variants were observed in *RAD52*, *FANCD2*, and *MRE11*; however, all were observed at >1% in population variation databases and were, therefore, less likely to be functionally detrimental.

### 3.6. Genetic Similarity

Hispanic/Latino populations are genetically heterogeneous and share genetic similarities with European, Indigenous American, and African populations in varying proportions. Genetic similarity to the reference populations of the 1000 Genomes Project was assessed using common germline single nucleotide polymorphisms (SNPs). The patients in the PR H/L cohort exhibited a range of similarity proportions predominantly to European, African, and Indigenous American populations. The proportions were similar to those observed in Puerto Rican Southern Principalities, although wide standard deviations were noted in the small PR H/L cohort (Supplemental Table S7). Multidimensional scaling analysis showed a high degree of overlap between the genetic similarity proportions of the PR H/L PCa cohort and individuals from Puerto Rico participating in large population genetics studies (Supplemental Figure S7). Similar to other studies, individuals from Puerto Rico (including the PR H/L cohort) tended to be more similar to African populations.

### 3.7. Differential Gene Expression Based on Gleason Category

**Figure 3:**
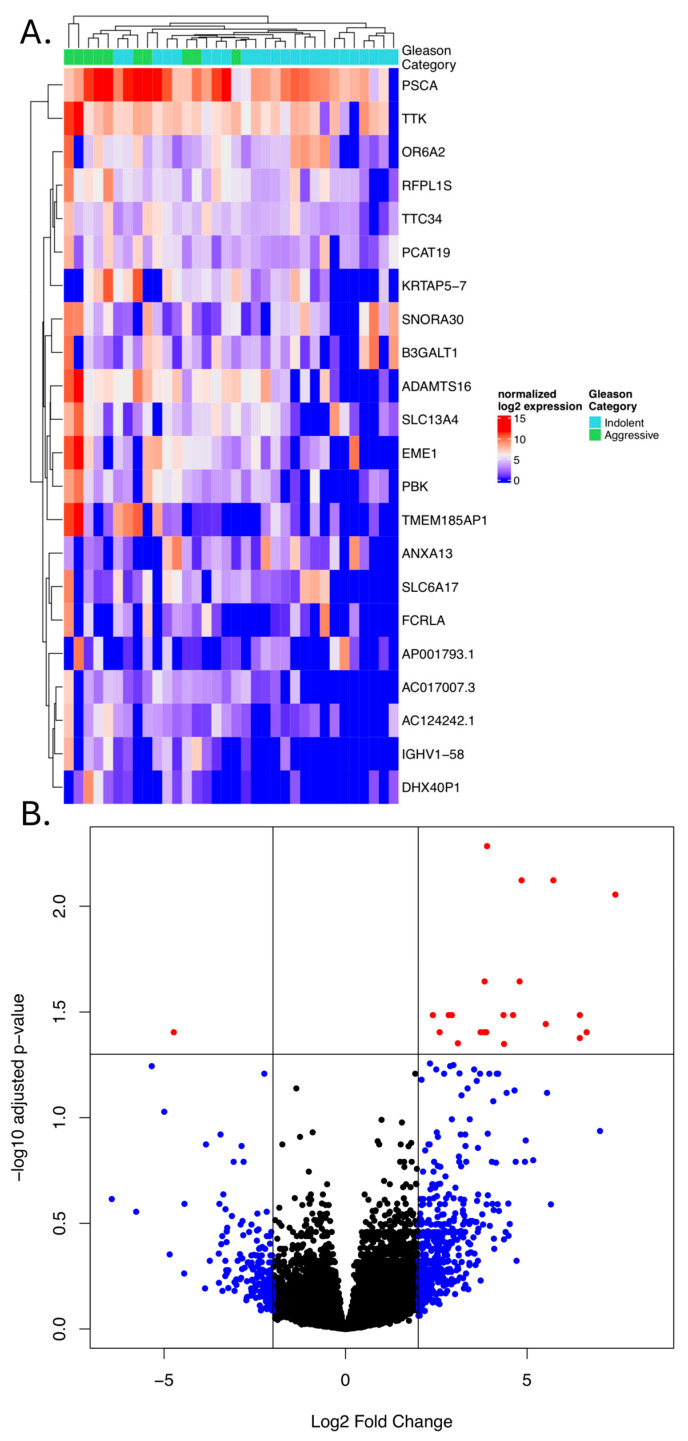
Comparison of gene expression in 34 prostate cancer patient tumors with indolent and aggressive Gleason categories. (**A**) Heatmap: The x-axis denotes gene expression in each sample, and the y-axis lists significantly differentially expressed genes (*p* < 0.05, log2 fold-change > 2). (**B**) Volcano plot: gene expression Log2 fold change in aggressive vs. indolent is shown on the x-axis, −log10 adjusted *p*-value on the y-axis. Red datapoints pass both *p*-value and fold change criteria.

Differential gene expression between Gleason category indolent and aggressive tumors was compared using RNA sequencing (Supplemental Tables S8 and S9). A total of 21 genes were more highly expressed in aggressive tumors and one showed decreased expression (Figure 3, Supplemental Figure S8). *ANXA13* was the only gene with decreased expression in the aggressive group. Consistently upregulated genes across the aggressive group include *TTC34*, *TTK*, *PCAT19*, *RFPL1S*, *SLC13A4*, *OR6A2*, *ADAMTS16*, *SNORA30*, *PBK*, *PSCA*, *AC124242.1*, *KRTAP5-7*, *AC017007.3*, *EME1*, *B3GALT1*, *AP001793.1*, *SLC6A17*, *DHX40P1*, *IGHV1-58*, *TMEM185AP1*, and *FCRLA*. All genes displayed expression overlap between groups, but samples of the same Gleason category tended to group together based on unsupervised clustering (Figure 3). Pathway enrichment analysis was performed using enrichR (accessed February 2026), which suggested enrichment in related GO Biological process 2025 “Response to DNA Damage Checkpoint Signaling” (Fisher’s exact adj. *p* = 0.084; *EME1*), MSigDB Hallmark 2020 “G2-M Checkpoint” (Fisher’s exact adj. *p* = 0.081; *PBK*, *TTK*), and KEGG 2026 “Homologous Recombination” (Fisher’s exact adj. *p* = 0.095; *EME1*). All of these genes were more highly expressed in aggressive tumors.

We further explored co-expression patterns with the androgen receptor (*AR*) by dividing samples into high and low *AR* expression groups (median log2 expression cut-off point: 12.61) and performing differential expression analysis. Six differentially expressed genes were identified between tumors with low and high AR expression (Supplemental Figure S9). *PRSS2*, *SLC2A2*, and *SEMG1* were downregulated in AR-high tumors, whereas *BCDIN3D*-*AS1*, *HERC2P5*, and *GABRA1* were upregulated.

## 4. Discussion

This study is the first to report tumor sequencing in Puerto Rican H/L men with PCa. Despite the high PCa mortality rate, there is a paucity of published genomic data from this population group. The causes of the high PCa mortality rates (26.7 per 100,000) among Puerto Rican men compared to US H/L (16.2 per 100,000) and non-Hispanic white (NHW) (18.2 per 100,000), as reported by Miller et al. [66], are not completely understood. Del Pino et al. [11] evaluated the differences in PCa characteristics among H/L from different countries of origin using the National Cancer Database. Puerto Rican H/L (PR H/L) men had the worst overall survival (HR = 1.27, CI = 1.18–1.47, multivariable), compared to Mexican Americans. PR H/L patients (n = 642) who were treated at the Veterans Administration Caribbean Healthcare System (VACHS, San Juan, PR, USA) had a higher risk of biochemical recurrence (BCR) (Hazard Ratio (HR) = 1.27, *p* < 0.001), metastases (HR = 1.49, *p* = 0.014), castrate-resistant PCa (CRPC) (HR = 1.80, *p* = 0.001), and PCa-specific mortality (PCSM) (HR = 1.74, *p* = 0.011) [12].

Most genomic studies have predominantly focused on individuals of European ancestry, with H/L populations representing approximately 1% of the patients in genome-wide association studies [19], 3.5% of all TCGA patients, and only 1.4% of TCGA-PRAD patients (NCI Genome Data commons, accessed October 2025). This lack of representation poses challenges in understanding the genomic factors influencing PCa risk among H/L men. In a study of non-Hispanic U.S. Veterans with metastatic PCa, NHW men were more likely to have somatic alterations in the AKT/PI3K pathway, AR axis, and various tumor suppressor genes than NHB men, who had more *SPOP* mutations [67]. Among Hispanic White men who contributed to the GENIE cohort at the Memorial Sloan Kettering Cancer Center, alterations in *TMPRSS2* and *ERG* were more common than those in NHW men. In metastatic tumors, alterations in *KRAS* and *CCNE1* were more frequently observed in Hispanic White men than in NHW men [39]. Large recent genomics studies have also provided insight into PCa evolutionary trajectory: the timing of driver alteration appearance was shown to impact disease progression. The link between genetics and tumor biology across populations has also been recently reported, although H/L populations still remain underrepresented [37].

To address the lack of genomic data on PCa tumors from PR H/L men, we undertook a pilot study of genomic characterization in this population. We performed preliminary comparisons of somatic mutation characteristics in PR H/L patients with primary low Gleason grade tumors in MSK-IMPACT (n = 315, H/L incidence 3–5%) and TCGA (n = 167, ethnicity not available or largely missing). We observed fewer mutations in the commonly mutated PCa genes *SPOP* and *TP53*, although these results were not significant. Mutation rates in the PR H/L cohort were significantly elevated in *E2F3* and *PRSS1*. Increased mutation frequency was observed in *KMT2C* but this was not significant. Truncations and deletions in *PTEN* mutations were observed in 5% of PR patients compared to 8–10% in MSK-IMPACT and TCGA. Overall, we observed similarities in the mutation signatures compared to TCGA [68], including the prevalence of clock-like and ROS patterns. We also noted chemotherapy signatures in some patients that were not commonly observed in TCGA. Recurrent CNVs are often clustered in larger regions. Deletions were clustered at chromosome ends, suggesting telomere shortening. Telomere length differences have been observed in prostate cancer and the ratio of tumor to normal telomere lengths has been associated with biochemical recurrence [69]. Further studies in all populations are important to determine the role of telomere length in PCa biology and clinical outcomes.

ETS family gene fusions are among the most common genomic alterations in PCa. We observed a significant decrease in such fusions: 12% in PR vs. 34% in MSK-IMPACT and 57% in TCGA. Although we observed copy number amplifications via whole-exome sequencing in patients (a fusion or amplification in an ETS family gene was observed in 25% of patients), the functional consequence is unclear: *ERG* expression was increased in tumors with *TMPRSS2-ERG* fusions but not in a tumor with *ERG* amplification. In contrast to our study, Arenas-Gallo et al. observed a higher proportion of *TMPRSS2/ERG* alterations (including fusions, copy number variations, and point mutations) in H/L men (40–50%) [39]. The limited sample size of the H/L cohorts makes comparison challenging, and additional samples are needed to confirm these preliminary observations as well as differences across diverse H/L populations. In addition, it is possible that technical aspects of our study (FFPE preserved tumors) limited the sensitivity to detect *TMPRSS2/ERG* fusions. We observed a higher level of *ERG* expression in tumors with a *TMPRRS2/ERG* fusion, but also observed two additional tumors with high *ERG* expression and no detected fusion, suggesting that the PR H/L cohort may have up to 18% of tumors with overexpressed *ERG*.

Overall, our observations of the decreased incidence of certain common PCa somatic alterations raise questions regarding the additional driver events that may contribute to PCa development in Puerto Rican men. Although we observed a trend of higher TMB with higher pathological grade and aggressive Gleason category, this association was not significant, as power was limited by cohort size and the lower number of somatic events in PCa. Further studies are required to confirm these results and better understand the factors that drive aggressiveness in this group.

Only one rare truncating germline variant in the DNA damage repair genes was observed in our cohort: *BRCA2*: p.K3326*. The role of this mutation remains controversial. This has been suggested to be pathogenic (reviewed in [70]). In one study, no association with PCa was observed, although associations with breast and ovarian cancers were observed [71]. Interestingly, all six patients in TCGA with truncated *BRCA2* germline variants also had K3326*. Although the incidence in our dataset was higher than that in TCGA, this difference was not significant, likely because of limited power. As of this writing, *BRCA2* p.K3326* is classified as “Benign” in ClinVar with a two-star review status (criteria provided, multiple submitters, no conflicts). Although rare germline variants are important in PCa development and progression, further studies are needed to determine their role and incidence in the PR H/L population.

We examined the differences in gene expression between indolent and aggressive PCa in PR H/L men. The biological functions of the 21 genes observed to be upregulated in Gleason aggressive PCa can be broadly classified into seven categories: cell cycle and proliferation regulators, oncogenes and tumor promoters, transport and signaling, extracellular matrix and proteolysis, immune response and inflammation, olfactory receptors, and other diverse functions. Cell proliferation and cell cycle regulation genes, such as *TTK*, *PBK*, and *EME1*, are often associated with cell cycle progression and DNA replication/repair, which are critical for the rapid cell division characteristic of aggressive cancers. *TTK* (Threonine Tyrosine Kinase) and *PBK* (PDZ-binding kinase) are key kinases involved in cell cycle regulation and mitosis. *TTK* upregulation often indicates increased cell proliferation and genomic instability, both of which are frequently observed in aggressive cancers. *PBK*, a serine/threonine protein kinase that plays a role in the G2/M phase of the cell cycle, has been observed to be overexpressed in cancers and has been associated with increased aggressiveness of PCa [72]. *EME1* (Essential Meiotic Structure-Specific Endonuclease) plays a role in DNA repair and replication, particularly homologous recombination. The upregulation of these genes is consistent with increased cell cycle activity in more aggressive tumors. Prostate Stem Cell Antigen (*PSCA*) codes for a cell surface glycoprotein expressed in normal prostate tissue. It is involved in cell adhesion, proliferation, and differentiation; is frequently overexpressed in PCa and other cancers; and is associated with disease progression. *PSCA* has been proposed as a biomarker and target for cancer therapy [73]. Genes such as *SLC13A4* (Solute Carrier Family 13 Member 4) and *SLC6A17* (Solute Carrier Family 6 Member 17) are involved in solute transport, which can affect cellular metabolism and nutrient uptake processes that are often altered in cancer cells. *SLC6A17* is involved in neurotransmitter and amino acid transport and has been reported to be overexpressed in PCa [74] and other cancers.

*ANXA13* (Annexin A13) was the only gene whose expression tended to be higher in indolent Gleason tumors. The role of annexins in cancer varies depending on the specific annexin family member and the tumor type. *ANXA13* has been observed to be downregulated in PCa but upregulated (and even prognostic) in other cancers [75].

Although genetic factors may be important in population-level prostate cancer disparities in Puerto Rico, one important limitation of our study was the limited ability to address non-genetic contributors. Socioeconomic position (SEP) is a primary driver of mortality in Puerto Rico [76]. Key systemic issues include reimbursement disparities, insurance limitations and cost of care [77]. Puerto Rico faces a critical shortage of specialists necessary for managing prostate cancer due to physician migration to the U.S. mainland, urologist shortages and an aging workforce. Access to care is heavily influenced by geography. Specialized oncology and radiotherapy services are concentrated in the San Juan Metropolitan Area, leaving rural residents with significant travel burdens [77,78]. Inadequate transportation and a lack of localized medical resources in rural municipalities are linked to later-stage diagnoses [13]. Extreme weather events, specifically Hurricanes Irma and Maria (2017), caused profound and lasting disruptions in oncology care. Disasters have extended the time between diagnosis and the start of treatment [15]. Other non-genetic contributors are related to lifestyle, behavioral and clinical factors. MetS syndrome affects 43.3% of Puerto Rican adults, with nearly 50% of individuals with elevated glucose levels and 49% exhibiting abdominal obesity [79]. Mistrust of the healthcare system, limited disease awareness, and poor patient–provider communication often prevent men from seeking or completing treatment [80]. A high burden of cardiovascular and metabolic disorders among Puerto Rican survivors complicates treatment and reduces health-related quality of life [79,81]. Finally, environmental exposures may have a role in excess in prostate cancer incidence among residents with polluted municipalities. An excess risk of 15% (95%CI: 1.10–1.19, *p* < 0.0001) was observed for PCa among males of contaminated sites compared to residents of non-contaminated sites [17].

Our study has a few additional limitations that may affect the interpretability of our observations. First, the number of samples in this cohort of PR H/L men with prostate cancer was small, limiting the power to detect significant differences and associations. Second, the number of Hispanic/Latino patients in other cohorts was also limited, which further decreased power. The low number of samples in all cohorts increases the risk of stochastic differences, and highlights the need for continued efforts in profiling tumors from all populations. Third, all tumor samples in the PR H/L cohort were derived from FFPE blocks, which adds a potential technical difference when compared to other studies using predominant fresh/frozen tissue. Despite these limitations, the initial results suggest potential differences in the molecular features of PCa tumors that may be important for understanding PCa biology in PR and H/L populations.

## 5. Conclusions

We performed genomic characterization of tumors from a cohort of Puerto Rican men with primary prostate adenocarcinoma. We observed differences in somatic alterations, including lower mutation frequencies of common alterations and higher mutation frequencies of additional genes. We identified gene expression differences between indolent and aggressive Gleason groups, providing insight into the potential biology underlying disease progression. Further efforts are needed study all patients with PCa to better understand disease progression.

## Figures and Tables

**Table 1:** Clinicopathological characteristics of the study cohort.

		All (n = 35)	WES Subset (n = 20)	RNA-Seq Subset (n = 34)
Age at diagnosis	Mean (SD) [range]	63.75 (5.75) [53–73]	63.65 (5.59) [53–71]	63.75 (5.75) [53–73]
	N missing	3	3	2
Stage % (n)	T2	68.57 (24)	75.00 (15)	67.65 (23)
	T3	31.43 (11)	25.00 (5)	32.35 (11)
	Missing	0.00 (0)	0.00 (0)	0.00 (0)
Lymph nodes % (n)	N0	91.43 (32)	95.00 (19)	91.18 (31)
	N1	5.71 (2)	5.00 (1)	5.88 (2)
	Missing	2.86 (1)	0.00 (0)	2.94 (1)
Distant metastasis % (n)	M0	34.29 (12)	35.00 (7)	35.29 (12)
	Missing	65.71 (23)	65.00 (13)	64.71 (22)
Gleason Grade % (n)	1	22.86 (8)	35.00 (7)	20.59 (7)
	2	48.57 (17)	45.00 (9)	50.00 (17)
	3	20.00 (7)	20.00 (4)	20.59 (7)
	4	2.86 (1)	0.00 (0)	2.94 (1)
	5	5.72 (2)	0.00 (0)	5.88 (2)
	Missing	0.00 (0)	0.00 (0)	0.00 (0)
Gleason category	Indolent	71.43 (25)	80.00 (16)	70.59 (24)
	Aggressive	28.57 (10)	20.00 (4)	29.41 (10)
	Missing	0.00 (0)	0.00 (0)	0.00 (0)
PSA	Mean (SD) [range]	6.60 (5.43) [0–26]	7.30 (5.82) [0.016–26]	6.60 (5.43) [0–26]
	N missing	1	1	0

## Data Availability

Sequencing data and associated demographic information have been deposited in dbGAP/SRA under accession phs004487.v1.p1.

## Supplementary Materials

The following supporting information can be downloaded at: https://www.mdpi.com/article/10.3390/cancers18071091/s1. Figure S1. Comparison of the mutational frequencies in the Puerto Rico cohort with the Memorial Sloan Kettering (MSK) and TCGA Pan Cancer Atlas. MSK and TCGA are restricted to primary tumors and include all GS groups. Fisher’s exact test *p*-value < 0.05 for PR H/L vs. MSK-IMPACT noted with *; vs TCGA noted with +. Figure S2. Gene coverage: fraction of bases in each gene covered by at least 10 reads Figure S3. Oncoprint of recurrent somatic Copy Number Variants. Squares are colored by event type (deletion: blue, amplification: red). Samples are grouped by Gleason Grade (aggressive: green, indolent: light blue) and then copy altered gene count. Genes are grouped by chromosome location. Figure S4. Gene expression grouped by CNV status. Copy neutral: 2, Amplification: 3. y-axis shows log2 normalized gene expression. Correlation statistics (Kendall’s method) are shown. Figure S5. Oncoprint showing Copy Number alternations and truncations in DNA damage repair genes. A) Homologous Recombination Repair genes; B) Nucleotide Excision Repair genes. Amplifications are shown in red, deletions in blue, and truncating mutations in small white boxes. Figure S6. ERG expression vs alteration: Boxplot of log2 normalized ERG expression grouped by ERG alteration type. Amplification was not evaluated for significant difference as there was one sample. Figure S7. Multidimensional scaling of whole-exome sequenced (WES) prostate cancer patients (n = 20, “PUR_PCA”) in reference to selected populations from the 1000 Genomes Project (TGP). For visualization, subpopulations within European (EUR: CEU, GBR, FIN, TSI), African (AFR: YRI, LWK, GWD, MSL), and East Asian (EAS; CHB, JPT, CHS) continental groups were combined, and only the parent populations are shown. Admixed African (AFR: ACB, ASW) and American (AMR) populations are represented individually. The South Asian (SAS) population was not included to minimize visual complexity. Figure S8. Boxplots detailing gene expression divided by Gleason group. Normalized log2 expression is shown on y-axis. Figure S9. Boxplots detailing gene expression divided by AR expression group. AR log2 expression groups defined as high: >12.61, low <=12.61. Normalized log2 expression is shown on y-axis. Table S1. WES germline QC: sequence quality metrics from germline whole exome sequence samples. Table S2. WES tumor QC: sequence quality metrics from tumor whole exome sequence samples. Table S3. Somatic Mut QC: mutation counts and metrics following somatic mutation detection. Table S4. Recurrent CNVs: genes and chromosomal locations of recurrent CNVs. Table S5. HRR NER Genes: lists of Homologous Recombination Repair (HRR) and Nucleotide Excision Repair (NER) genes. Table S6. Germline Var QC: variant counts and metrics following germline variation detection. Table S7. Genetic similarity: Mean genetic similarity compared to continental populations. Table S8. RNAseq align: RNA sequencing alignment metrics. Table S9. collectRNAseq: additional metrics related to RNA sequencing quality. Reference [82] is cited in Table S7.
