# Supplementary material for: Genomic Analysis of Puerto Rican Hispanic/Latino Men with Prostate Cancer
Source: Cancers (Basel). 2026 Mar 27;18(7):1091. doi: 10.3390/cancers18071091 (PMC13072284; doi:10.3390/cancers18071091)

Supplemental Figure 1: Comparison of the mutational frequencies in the Puerto Rico cohort with the Memorial Sloan Kettering (MSK) and TCGA Pan Cancer Atlas. MSK and TCGA are restricted to primary tumors and include all GS groups. Fisher's exact test p-value <0.05 for PR H/L vs. MSK-IMPACT noted with *; vs TCGA noted with +.

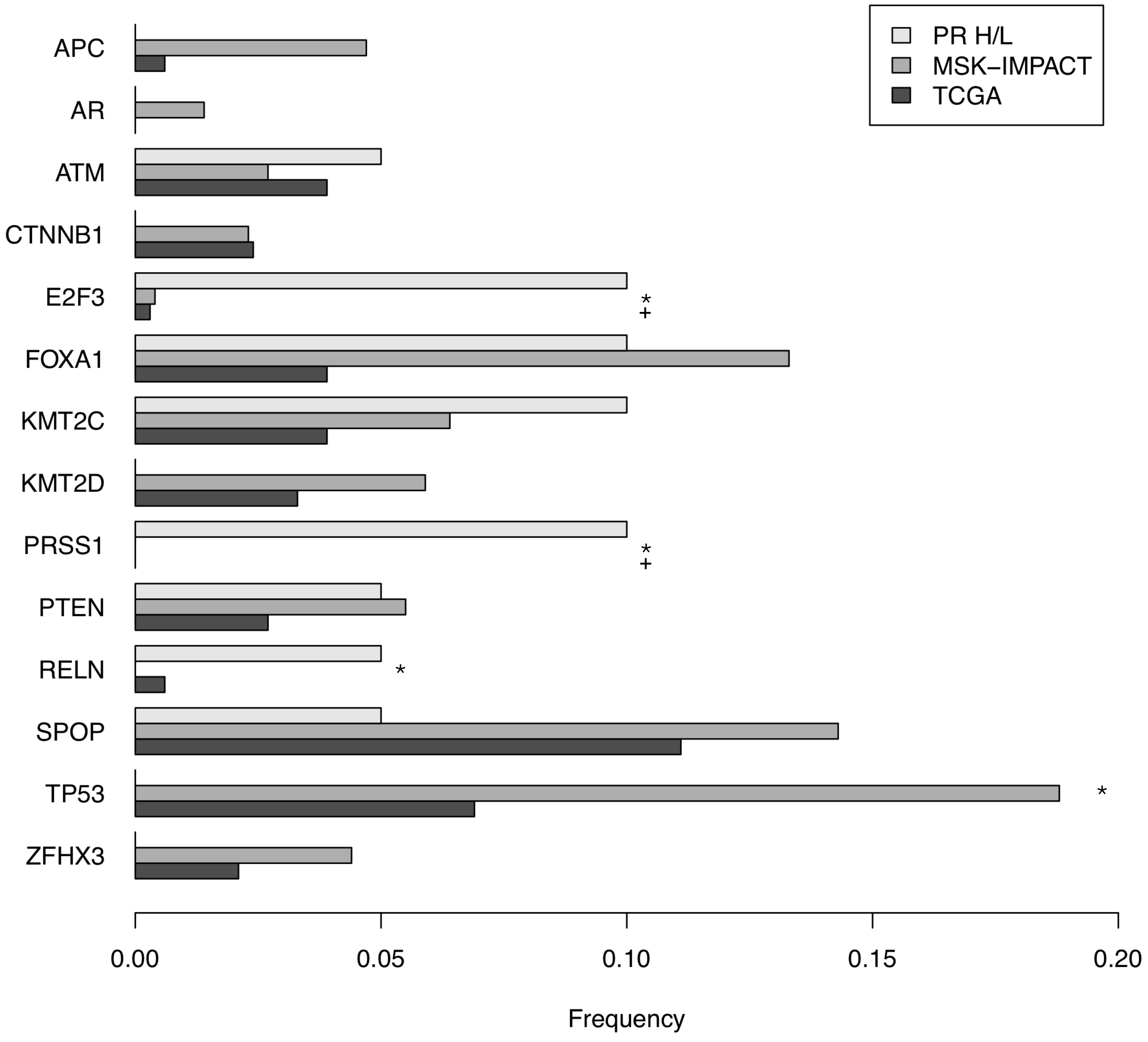

Supplemental Figure 2 – Gene coverage: fraction of bases in each gene covered by at least 10 reads

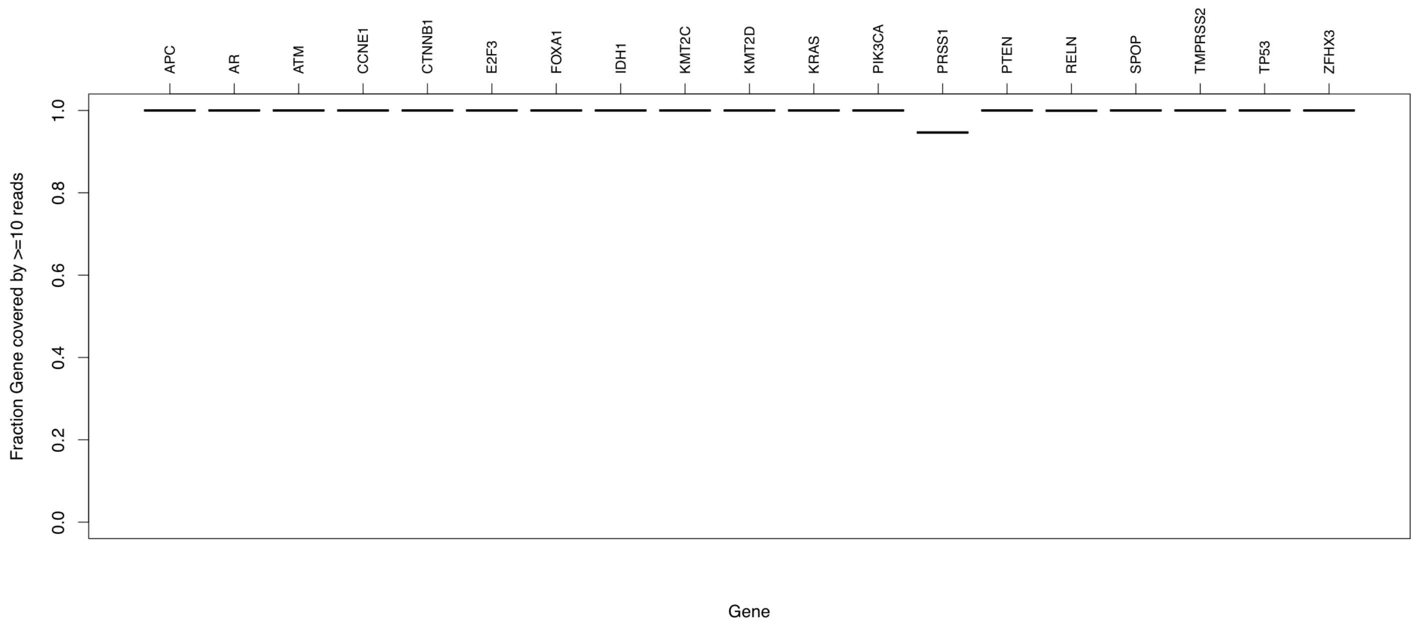

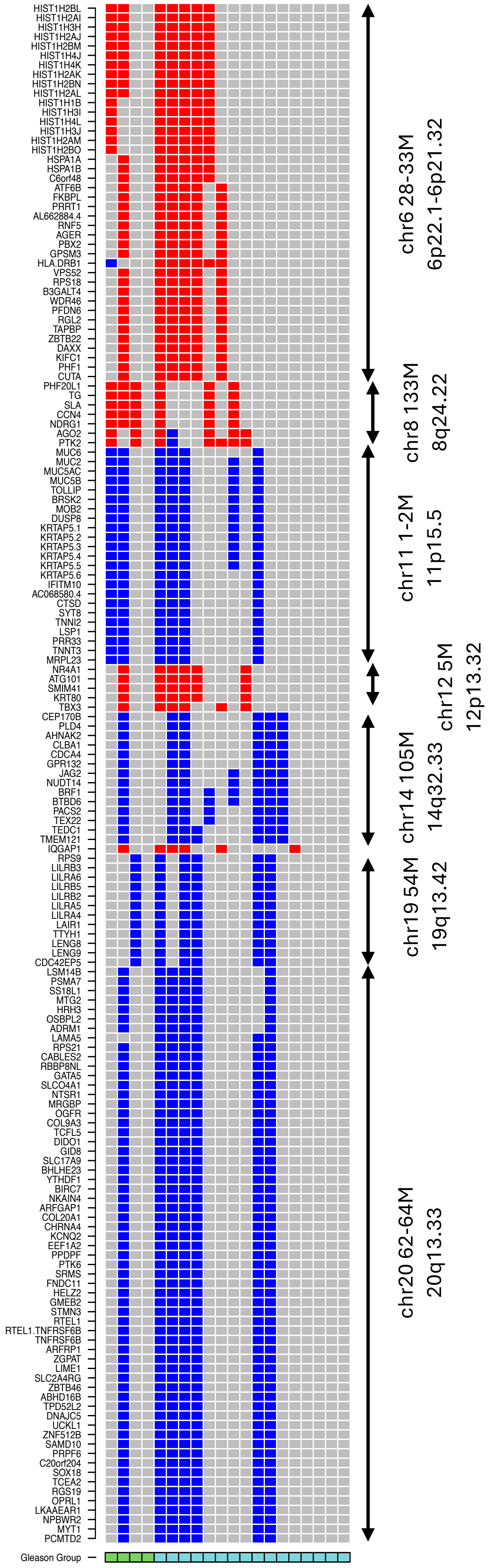


Supplemental Figure 3 – Oncoprint of recurrent somatic Copy Number Variants. Squares are colored by event type (deletion: blue, amplification: red). Samples are grouped by Gleason Grade (aggressive: green, indolent: light blue) and then copy altered gene count. Genes are grouped by chromosome location.

Supplemental Figure 4 – Gene expression grouped by CNV status. Copy neutral: 2, Amplification: 3. y-axis shows log2 normalized gene expression. Correlation statistics (Kendall's method) are shown.

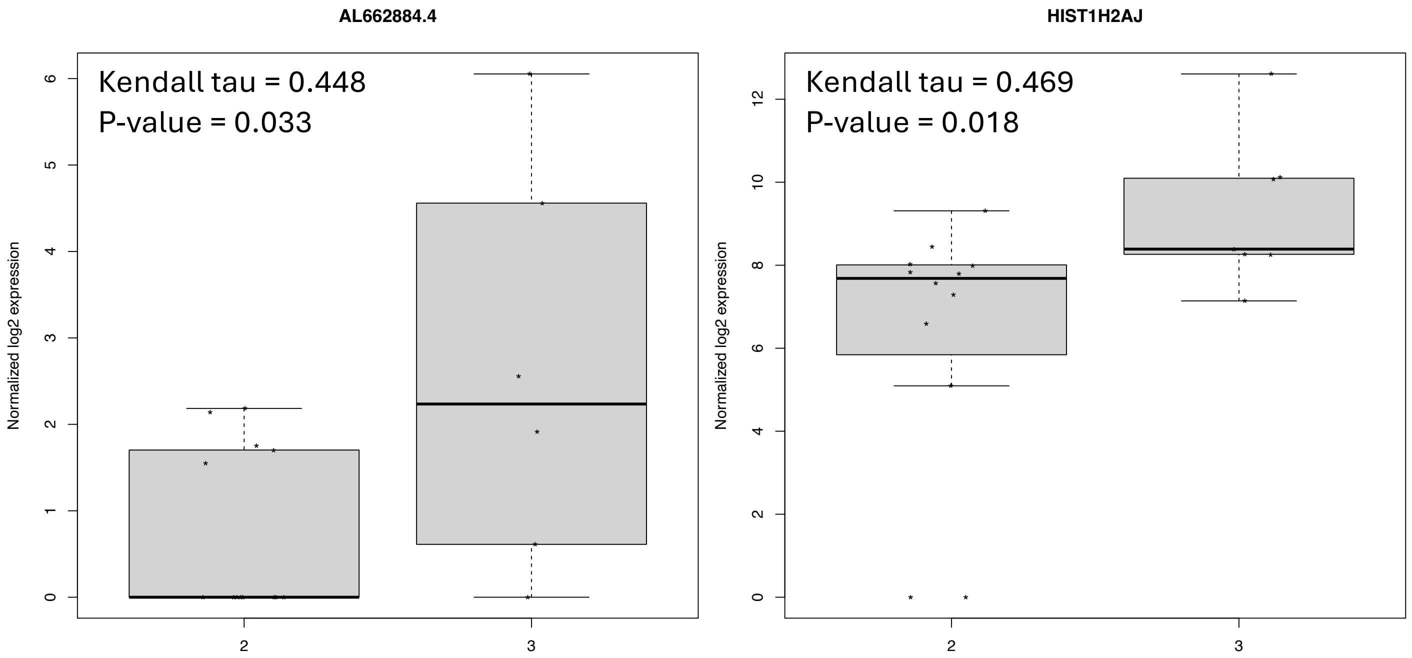

Supplemental Figure 5: Oncoprint showing Copy Number alternations and truncations in DNA damage repair genes. A) Homologous Recombination Repair genes; B) Nucleotide Excision Repair genes. Amplifications are shown in red, deletions in blue, and truncating mutations in small white boxes.

A.

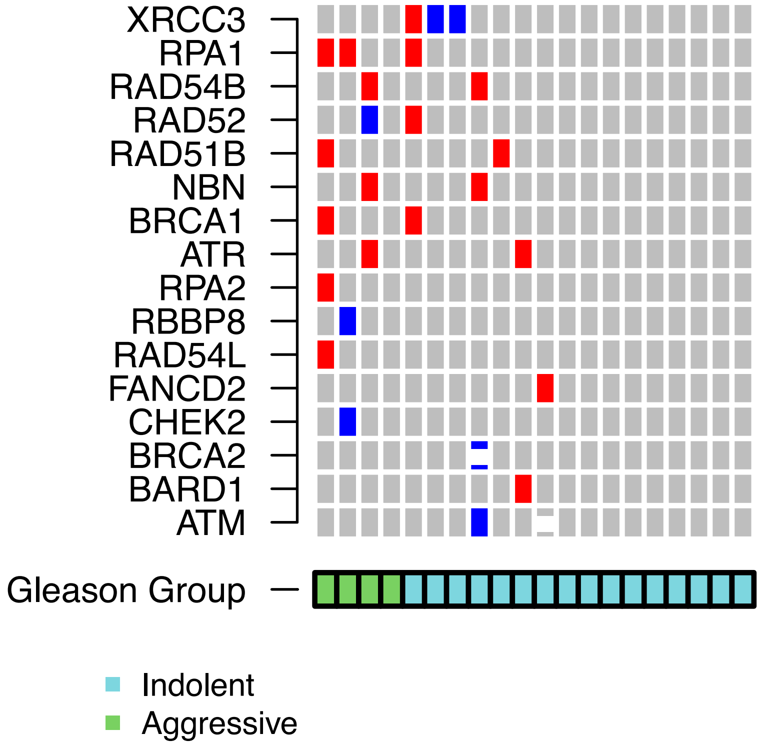


B.

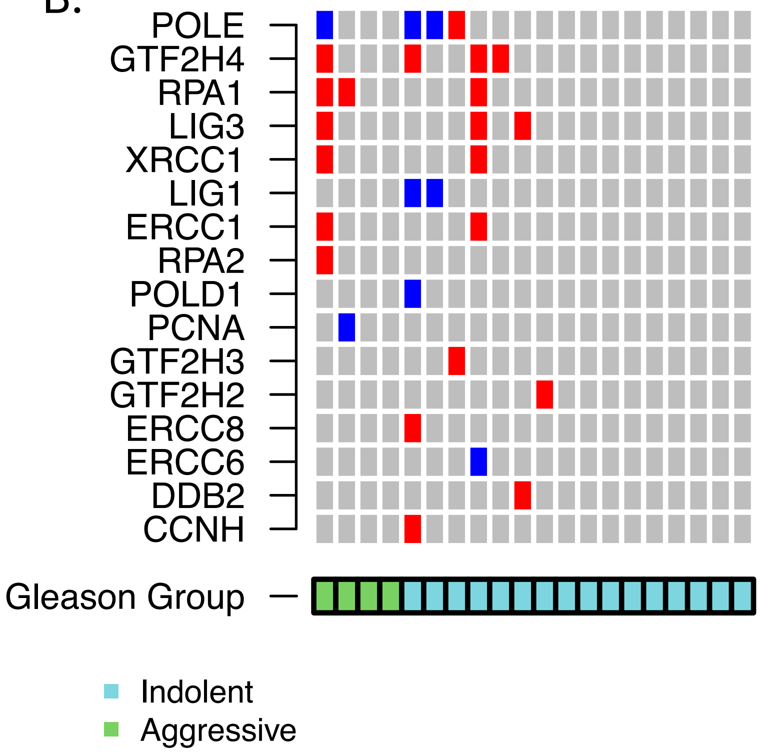

Supplemental Figure 6: ERG expression vs alteration: Boxplot of log2 normalized *ERG* expression grouped by *ERG* alteration type. Amplification was not evaluated for significant difference as there was one sample.

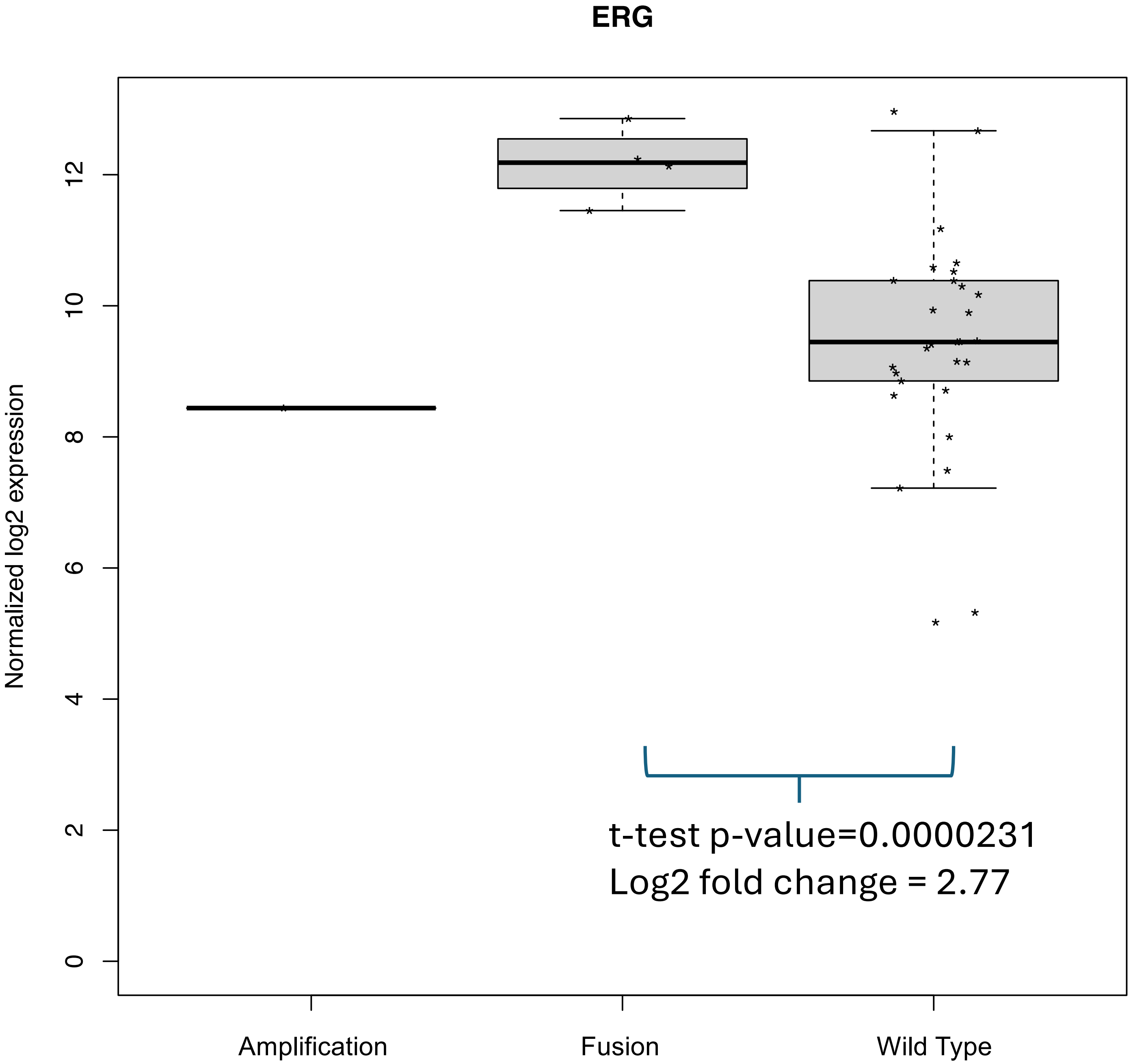

Supplemental Figure 7.
Multidimensional scaling of whole-exome sequenced (WES) prostate cancer patients (n = 20, “PUR_PCA”) in reference to selected populations from the 1000 Genomes Project (TGP). For visualization, subpopulations within European (EUR: CEU, GBR, FIN, TSI), African (AFR: YRI, LWK, GWD, MSL), and East Asian (EAS; CHB, JPT, CHS) continental groups were combined, and only the parent populations are shown. Admixed African (AFR: ACB, ASW) and American (AMR) populations are represented individually. The South Asian (SAS) population was not included to minimize visual complexity.

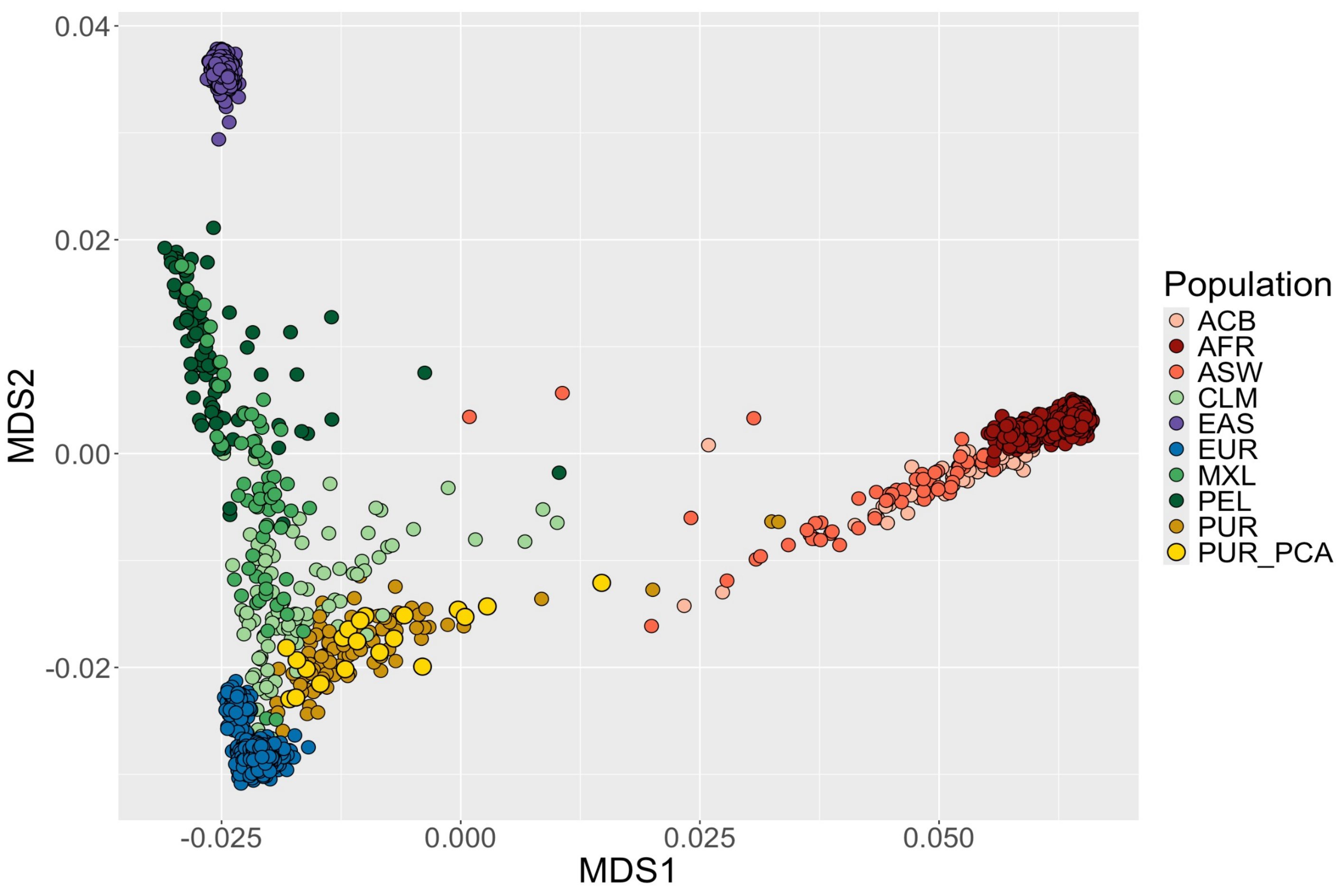

Supplemental Figure 8: Boxplots detailing gene expression divided by Gleason group. Normalized log2 expression is shown on y-axis.

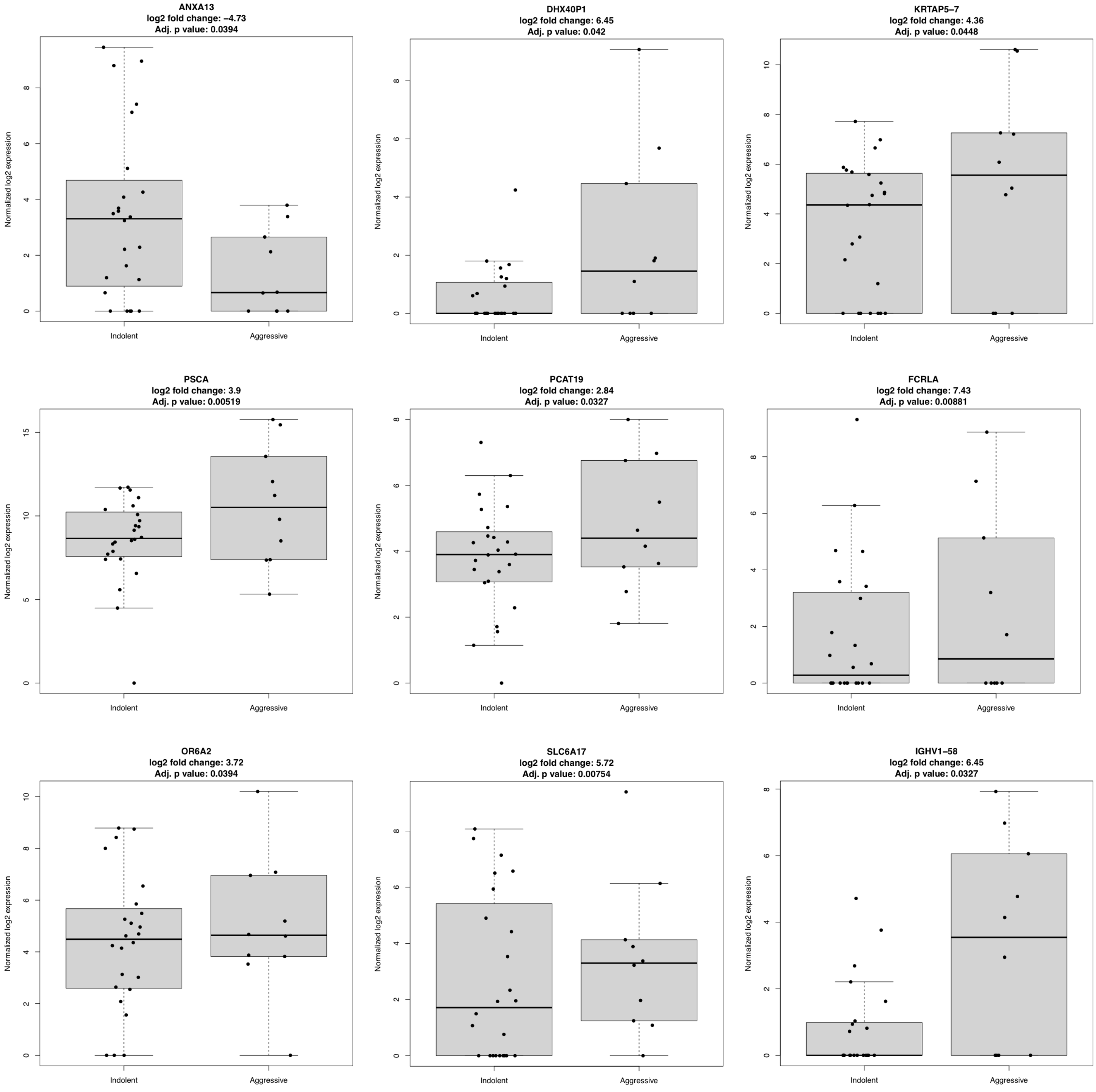

Supplemental Figure 8 continued: Boxplots detailing gene expression divided by Gleason group. Normalized log2 expression is shown on y-axis.

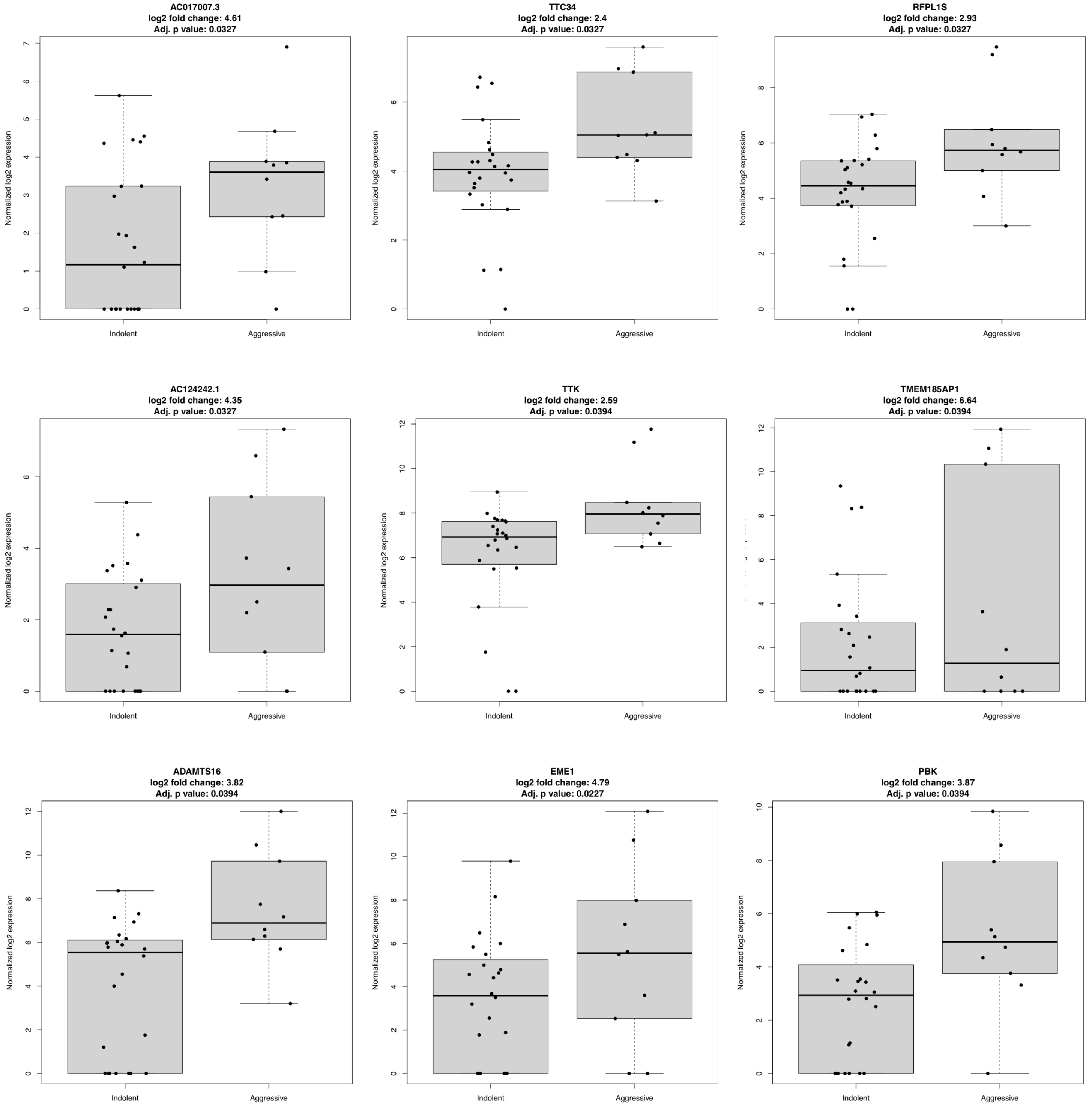

Supplemental Figure 8 continued: Boxplots detailing gene expression divided by Gleason group. Normalized log2 expression is shown on y-axis.

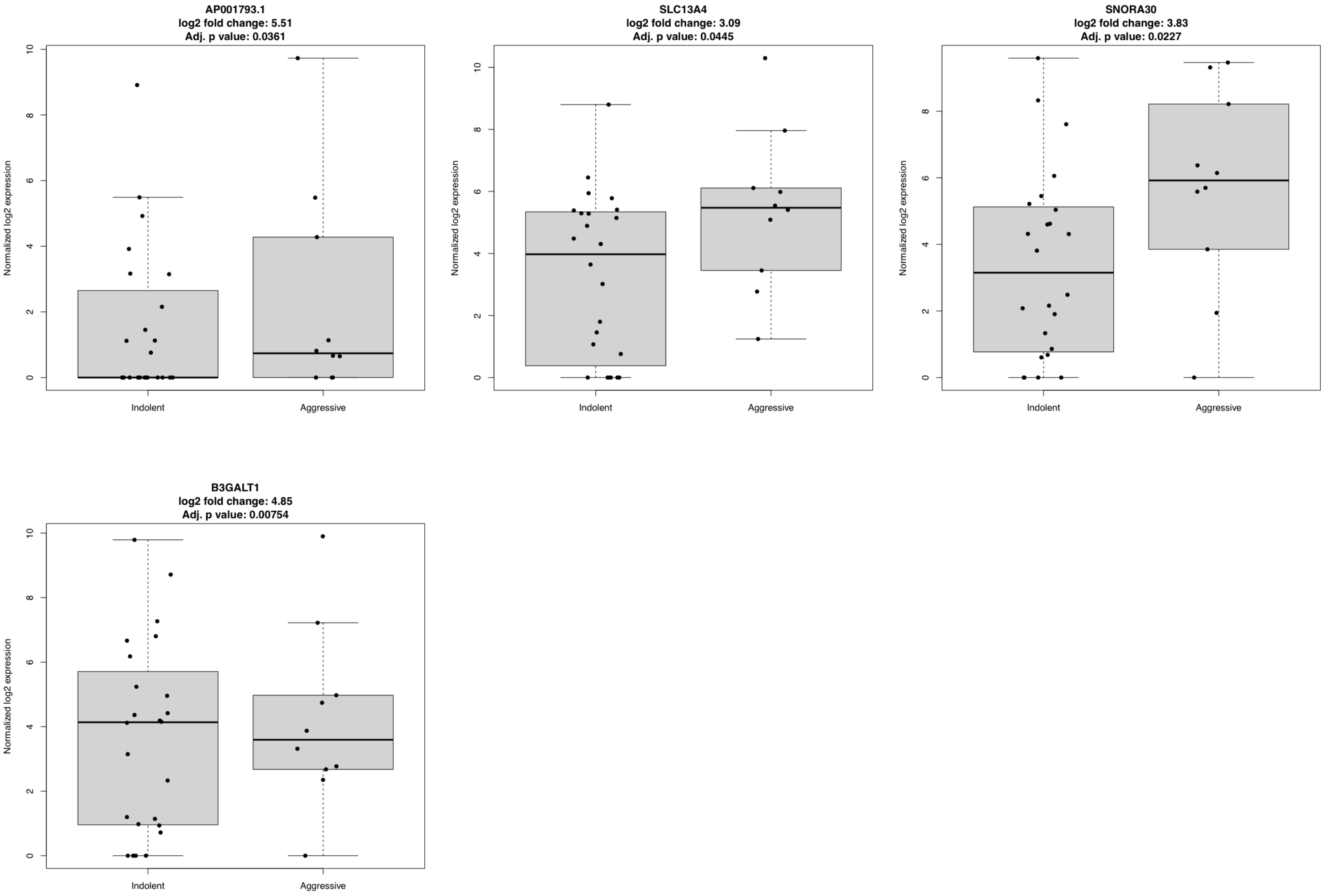

Supplemental Figure 9: Boxplots detailing gene expression divided by AR expression group. AR log2 expression groups defined as high: >12.61, low <=12.61. Normalized log2 expression is shown on y-axis.

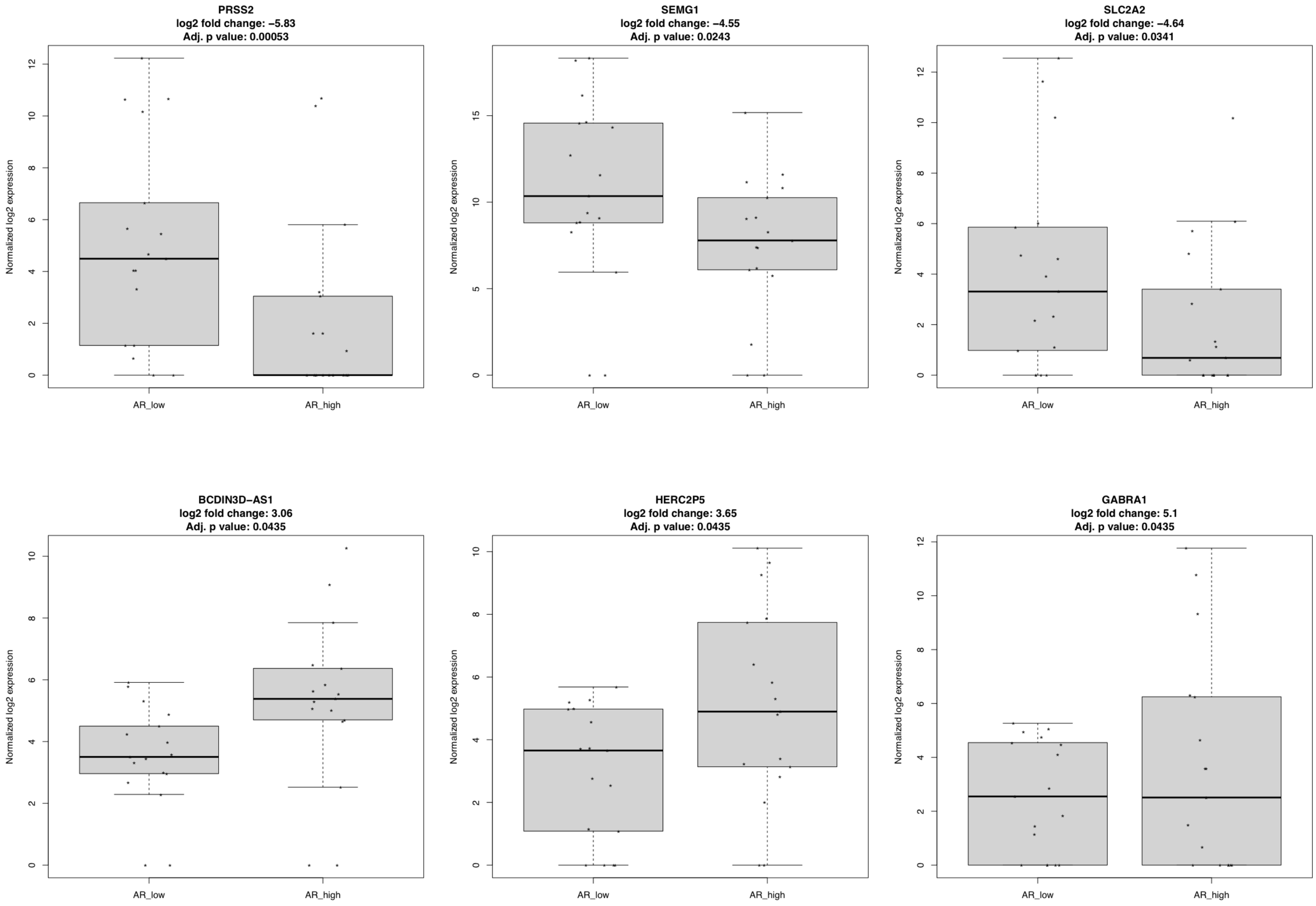

Supplement: Supplementary file 1 [file cancers-18-01091-s001.zip › Supplemental_Figs.pdf]
